# MicroRNA–target interactions: new insights from genome-wide approaches

**DOI:** 10.1111/j.1749-6632.2012.06745.x

**Published:** 2012-10-10

**Authors:** Dooyoung Lee, Chanseok Shin

**Affiliations:** Department of Agricultural Biotechnology, Seoul National UniversitySeoul, Republic of Korea

**Keywords:** microRNA, target recognition, genome-wide approach

## Abstract

MicroRNAs (miRNAs) are key posttranscriptional regulators of gene expression involved in diverse biological pathways in bilateral animals and plants. The key to understanding the biological function of a miRNA is to identify its regulatory targets. Although a few miRNA targets have been identified genetically, the rapidly expanding list of miRNAs has necessitated genome-wide tools for identifying target mRNAs, and a number of computational and experimental approaches have consequently emerged. Some of these approaches have also provided insights into the mechanistic aspects of miRNA-mediated regulation, another intensely debated area in the miRNA field. Here, we review several emerging features of miRNA–target interactions in animals and genome-wide approaches for probing those interactions.

## Introduction

The first microRNA (miRNA), *lin-4*, was discovered in 1993 through genetic screens in nematode worms.^1^,^2^ It took seven years for the second miRNA, *let-7*, to be found, also in worms.^3^ Soon after the observation that the *let-7* miRNA is evolutionarily conserved among a wide range of bilateral animals,^4^ efforts were made by several laboratories to identify additional miRNA genes in the genomes of worms, flies, and mammals.^5^–^7^ The number of known miRNAs has since escalated at an exponential pace, with the aid of concurrent advances in sequencing technologies and computational biology, and over 20,000 mature miRNA sequences from over 160 species have been deposited in the miRNA database (miRBase, release 18) at the time of writing.^8^

By virtue of intensified efforts over the past two decades, much is now known about the genomics, biogenesis, mechanisms, and biological functions of this class of tiny regulatory RNAs.^9^–^13^ The miRNA genes are transcribed into long primary transcripts containing local stem–loop structures, which are sequentially processed by the action of RNase III-type enzymes. Of the resulting ∼22-base pair miRNA duplex, one strand stably associates with an Argonaute (Ago) protein to form a functional miRNA-induced silencing complex (miRISC). The mature miRNA guides the miRISC to target mRNAs through base-pairing and directs posttranscriptional repression, the mechanism of which is largely dependent on the extent of base-pairing complementarity between the two nucleic acids. At sites with near-perfect complementarity, as shown in the pairing of most plant miRNAs with their targets, miRNAs can trigger Ago-catalyzed endonucleolytic cleavage of target mRNAs. In animals, however, miRNA–target pairing is often limited to a short stretch of the miRNA sequence and usually leads to translational repression, mRNA decay, or both. Nearly every major biological pathway in bilateral animals appears to be under the control of miRNAs, including developmental timing, cell differentiation and proliferation, apoptosis, energy metabolism, and antiviral defense.

The major key to understanding the biological function of an miRNA is to identify its regulatory targets. For a few of the initially identified miRNAs, such as *lin-4* and *let-7* in worms, their regulatory targets and presumable functions in larval development were already demonstrated by genetic analyses even before their molecular identity was uncovered.^14^,^15^ However, the rapid expansion of the catalog of miRNAs has necessitated genome-wide tools for identifying target mRNAs, and a number of computational and experimental approaches, often complementary to each other, have been developed and revised concomitantly. Some of these have not only captured miRNA targets but also have provided further insights into the mechanism of miRNA-mediated regulation, another controversial issue in the miRNA field.

In this review, we discuss several key features of miRNA–target interactions in animals in light of recent findings. We also introduce a variety of tools for genome-wide probing of miRNA–target interactions. Finally, we briefly summarize the current understanding of the mechanistic aspects of miRNA-mediated regulation.

## Target recognition by microRNAs: the seed rule and beyond

The prevailing model for miRNA-target interactions in animals was initially hinted at by the interaction of the *lin-4* miRNA with its target mRNA, *lin-14*: *lin-4* binds to multiple conserved sites within the 3′ untranslated region (UTR) of the *lin-14* mRNA with partial base-pairing complementarity.^1^,^2^ One notable feature in this founding example was that complementary sites in the *lin-14* 3′ UTR possess ∼8-nt “core elements” that match to the 5′ region of *lin-4*.^2^ Indeed, the presumable importance of the 5′ region of miRNAs began to emerge with the observation that it tends to be most evolutionarily conserved,^16^ and it frequently has perfect complementarity with 3′ UTR elements that are responsible for posttranscriptional repression of certain mRNAs in flies.^17^

**Figure 1:**
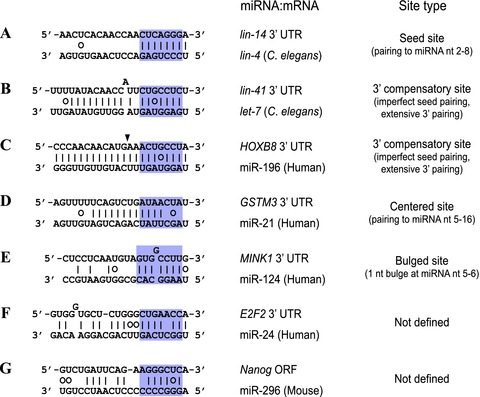
Examples of miRNA–target interactions. Pairing schemes of several miRNA–target interactions are illustrated. The miRNA seed region is shaded in purple. Watson–Crick pairs are indicated by solid lines and G:U wobble pairs by open circles. (A) The represented *lin-4* site in the *C*. *elegans lin-14* 3′ UTR exemplifies canonical seed sites, which exhibit perfect and contiguous pairing to the miRNA nucleotides 2 to 8. (B and C) 3′ compensatory sites display substantial pairing to the 3′ region of the miRNA to compensate for a single-nucleotide bulge or G:U wobble pair in the seed region. For the miR-196 site in mammalian *HOXB8*, the supplementary pairing is extensive enough for the miRNA to direct Ago2-catalyzed cleavage of the target mRNA. The position of cleavage is indicated by an arrowhead. (D) Centered sites do not have a seed match but instead exhibit 11–12 contiguous pairing to the central region of the miRNA. (E) Bulged sites perfectly match to the miRNA seed region except for a single nucleotide bulged out at the position corresponding to the miRNA nucleotides 5-6. (F and G) Some experimentally validated target sites have peculiar pairing schemes difficult to be generalized.

The seed rule states that perfect and contiguous pairing to the 5′ region of the miRNA nucleotides 2–8, called the seed region, is crucial for the specificity of target selection (Fig. 1A). This concept was first introduced in early attempts at computational target prediction, wherein the seed region retrieved the largest number of conserved target sites above the background noise across the entire miRNA sequence.^18^ Soon after, direct experimental evidence for the broad scope of the seed rule came from large-scale transcriptomic and proteomic studies, which showed that overexpression or depletion of a miRNA induces detectable changes in the output of gene sets that are enriched in the corresponding seed matches.^19^–^23^ Subsequent biochemical and structural findings provided a better understanding of the seed rule in molecular terms. In the context of the effector complex, the seed region disproportionately contributes to the energy required for target binding,^24^,^25^ and, consistently, the crystallographic studies of bacterial or human Ago proteins bound to guide nucleic acids show that the seed region is preorganized in a helical conformation such that its bases are exposed and positioned for base pairing with the target RNA.^26^–^28^

The nucleotide immediately downstream of the seed site is often a conserved A,^29^ which is capable of pairing to the first nucleotide of the many miRNAs that begin with U.^6^ However, the preferential conservation of A across from position 1 is also observed within target sites for the minority of miRNAs that do not begin with U.^29^ Notably, transcriptomic and proteomic studies showed that for miRNAs that do not begin with U, the seed sites with an unpaired A across from position 1 are more effective than those with a complementary nucleotide across from this position, suggesting that pairing to the first nucleotide of the miRNA, if it occurs, has little or no consequence for targeting.^22^,^30^ This is consistent with the observation that introduction of a mismatch at position 1 is tolerated and even potentiates the enzymatic activity of the effector complex *in vitro*.^24^ Indeed, a wealth of structural studies on Ago proteins in complex with the guide–target duplex indicate that the first nucleotide of the guide strand is distorted and does not engage in base pairing with the target RNA.^31^–^33^

Nonetheless, it appears unlikely that seed pairing is sufficient for embracing all target recognition events. Imperfect seed pairing is sometimes compensated for by substantial pairing to the 3′ region of the miRNA, as in the cases of the *let-7* sites in nematode *lin-41* and the miR-196 site in mammalian *HOXB8* (Figs. 1B and 1C).^3^,^34^ Recently, centered sites have been described, which exhibit 11–12 contiguous base pairings to the central region of the miRNA without substantial pairing to either end (Fig. 1D).^35^ However, more difficult to elaborate are an increasing number of experimentally identified target sites that neither obey the seed rule nor have a pairing scheme specific enough to be generalized into defined site types (Figs. 1F and 1G).^36^–^38^

The recent advent of genome-wide biochemical approaches to target identification might help draw underlying commonalities from these unusual interactions. For example, genome-wide analysis of Ago-binding sites in mouse brain revealed that 27% of the identified sites are “orphans,” lacking a perfect seed match to the 20 most highly expressed miRNAs.^39^A subsequent search for enriched motifs within those orphan sites identified a novel class of target sites, called bulged sites (Fig. 1E).^40^ The bulged sites perfectly match to the miRNA seed if not for a bulged-out nucleotide corresponding to the miRNA nucleotides 5–6, with the bulge nucleotide being competent to pair to the miRNA nucleotide 6 or the “pivot” residue. It was proposed that the pivot-pairing ability of the bulge nucleotide confers a thermodynamic advantage during a hypothetical phase of transitional nucleation, where five consecutive nucleotides can match the miRNA nucleotides 2–6 for the competent bulge compared to only four consecutive nucleotides for any other noncompetent bulges. The degree of repression and evolutionary conservation seen with bulged sites is somewhat less than that of canonical seed sites. The biological significance of these sites remains to be corroborated by further studies.

## Factors influencing targeting efficiency

### Residence of target sites

Most animal target sites identified so far reside in the 3′ UTRs of target mRNAs. This positional bias might arise because we have primarily focused on 3′ UTRs to search for target sites, largely motivated by the *lin-4* precedent and *in silico* convenience of target prediction. Alternatively, the preferential localization of target sites in 3′ UTRs might have a functional basis, perhaps preventing the bound miRISC from being displaced by the translational machinery.^10^ Microarray data and conservation analysis showed that target sites tend to become more effective and more selectively maintained ∼15 nucleotides downstream of the stop codon, apparently outside the path of the ribosome.^41^ Further supporting this mechanistic explanation, abolishing the upstream stop codon to extend the open reading frame (ORF) of a reporter gene through the target sites significantly impaired the miRNA-mediated repression, which was restored by introducing a cluster of rare codons upstream of the target sites.^42^

In recent years, it has become evident that 3′ UTRs of mRNAs are considerably more dynamic than previously appreciated. For example, about half of human genes undergo alternative cleavage and polyadenylation to generate multiple transcripts differing in their 3′ UTRs.^43^ Given that 3′ UTRs provide fertile ground for miRNA target sites, alternative polyadenylation (APA) and the consequent alteration of the 3′ UTR length is likely to affect the regulation by miRNAs. Indeed, the impact of APA on miRNA targeting has been investigated in the cellular context of increased proliferation, where a general shortening of 3′ UTRs is observed.^44^,^45^ A genome-wide analysis of alternative 3′ UTR isoforms in activated T cells showed a global increase in the relative expression of mRNAs with shorter 3′ UTRs, which have in average only half the number of conserved target sites compared to the longer isoforms prevalent in resting T cells.^44^ Notably, mRNAs with shorter 3′ UTRs produced substantially more protein than did those with longer 3′ UTRs, in part through escape from the miRNA-mediated repression. A similar phenomenon was observed in cancer cells, where the frequent loss of miRNA target sites by APA contributes to oncogene activation without genetic alteration.^45^

Although the vast majority of target sites extensively investigated have been those located in 3′ UTRs, several studies indicate that a considerable amount of targeting occurs within the ORFs of target mRNAs. Differential miRNA expression retrieved gene sets that are targeted through ORFs, albeit less frequently, as well as those targeted through 3′ UTRs.^20^,^22^,^41^ Genome-wide mapping of Ago-binding sites in mammals revealed that a fourth to a half of the identified sites are located in ORFs.^39^,^46^ As most target prediction algorithms rely on evolutionary conservation to distinguish authentic target sites from the multitude of heptamer segments that would exhibit seed complementarity by chance,^18^ tackling ORFs for target site searches was somewhat challenging, where strong conservation has been imposed to preserve the codon usage. However, the difficulty has been relieved as more genomes have been sequenced: for example, a computational search for highly conserved 8-mer motifs within ORFs across 17 vertebrate genomes identified a set of putative target sites, three of which being the *let-7* sites in the *DICER* mRNA ORF.^47^ On the other hand, one study employed a pattern-based algorithm that neither depends on conservation nor imposes a 3′ UTR bias to show that several pluripotency genes in mice are targeted through their ORFs, often in a species-specific manner.^37^ Taken together, these findings emphasize the underestimated importance of ORFs as recipients of miRNA activity.

### Target site accessibility and multiplicity

The accessibility to miRNA target sites can have a profound effect on their efficacy.^48^,^49^ Indeed, sequestering target sites within stable secondary structures substantially reduced the miRNA-mediated repression, with effects comparable to those of single-nucleotide mutations disrupting seed pairing.^48^ Comprehensive analysis of 3′ UTR context indicated that target sites tends to favor AU-rich neighborhood and positions away from the middle of long UTRs, both of which are associated with generally unstructured regions.^41^ In addition to local structures of RNA, various RNA-binding proteins (RBPs) can influence site accessibility. For example, HuR, a ubiquitously expressed RBP involved in mRNA stability control, was found to antagonize the miR-122–mediated repression of human *CAT1* mRNA in stressed conditions by binding to the 3′ UTR.^50^ Interestingly, the broad scope of functional antagonism between miRNAs and HuR was recently demonstrated by genome-wide mapping of HuR-binding sites, which showed that over 75% of 3′ UTRs with Ago-binding sites are also enriched in HuR sites and that mRNAs with overlapping miRNA target sites and HuR sites do not respond as well as those with only miRNA target sites upon depletion of the miRNA.^51^ Similarly, the miR-430 sites in zebrafish *nanos1* and *TDRD7* are in close vicinity to the binding sites for Dnd1, which appears to reduce target site accessibility and thereby alleviates miRNA-mediated repression.^52^

Target site multiplicity seems to be a widespread means of achieving effective target repression and combinatorial regulation by animal miRNAs. In general, increasing the number of target sites confers greater efficacy of repression in a multiplicative manner.^41^ When two target sites are present in proximity to each other with an optimal spacing of ∼10–40 nucleotides, however, they often act synergistically.^41^,^53^

### Lessons from lousy microRNAs

Aiming to examine the general applicability of the seed rule, one study established a sensor system based on the *C*. *elegans* neuronal miRNA, *lsy-6*, and tested 14 predicted targets containing perfect seed matches to *lsy-6* in their 3′ UTRs.^54^ Surprisingly, only 1 of 14 predicted targets was efficiently repressed by *lsy-6*. Based on these unusually weak responses from most predicted targets, the study proposed that perfect seed pairing is not a reliable predictor for miRNA-target interactions.^54^ On the other hand, another study suggested that secondary structures of the nonresponsive 3′ UTRs might constrain the accessibility to target sites.^49^

One recent study showed that the solution is neither the unreliable seed-based targeting model nor the inaccessible 3′ UTR structure, but the miRNA itself: the *lsy-6* miRNA has unusually low targeting proficiency because of its weak predicted seed-pairing stability (SPS) and its high target-site abundance (TA).^55^ miR-23, a mammalian miRNA that shows a poor targeting proficiency in HeLa cells, also has weak SPS and high TA. Weak SPS would reduce the fraction of miRNAs that engage in seed pairing at a given concentration and high TA would dilute the effect on each target mRNA by titrating miRNAs. Indeed, a few nucleotide substitutions that bring these parameters closer to those of typical miRNAs imparted improved proficiency to *lsy-6* and miR-23, even though the sites were in their original UTR contexts. Based on the observation that such “lousy” miRNAs have few targets, it was proposed that designing siRNAs with weak SPS and high TA would minimize undesirable off-target effects.

## The impact of microRNAs on mRNA expression and evolution

Microarray analysis followed by introduction of the miRNAs into HeLa cells that normally do not express them revealed modest downregulation of more than 100 mRNAs, most of which contain the corresponding seed matches.^20^ These genes were those that are generally expressed at low levels in the tissue that normally expresses the introduced miRNA.^20^ Consistent with this observation, expression profiling of mRNAs with conserved target sites for several tissue-specific miRNAs showed that they are generally expressed at lower levels in the tissue expressing the miRNA than in other tissues.^56^ Remarkably, time course profiles of tissue differentiation showed that these conserved targets are often highly expressed in earlier differentiation before miRNA expression and then their levels gradually decrease as the miRNA accumulates.^56^ Clearly, differential expression of miRNAs provides the opportunity to further establish and fine-tune tissue-specific transcript profiles during cellular differentiation.

The small size of the seed region intrinsically retrieves the multitude of heptamer segments that potentially serve as target sites. Many computational algorithms rely on evolutionary conservation, often considered a hallmark of functionality, to search for target sites likely to be more biologically relevant. However, nonconserved sites still outnumber preferentially conserved ones by about ten to one,^29^,^57^ raising the question of whether these sites might also be functional. Indeed, a large fraction of nonconserved sites exerted specific repression as efficiently as conserved sites when placed in reporters.^56^ Expression profiling of mRNAs with nonconserved sites defined a notable pattern that relates spatial expression of these messages with that of the miRNA. Specifically, the nonconserved sites tend to be depleted in the 3′ UTRs of genes that are highly and specifically expressed in the same tissue as the cognate miRNA.^56^ Such depletion appears to reflect the evolutionary pressure for tissue-specific mRNAs to avoid emergence of target sites for coexpressed miRNAs—a phenomenon known as selective avoidance.^56^ Consistent with this notion, the 3′ UTRs of housekeeping genes in animals are substantially shorter compared to those of other genes, plausibly avoiding fortuitous complementarity to a plethora of miRNAs they are confronted with in the cells.^58^ Given that the number of mRNAs under selective pressure to avoid targeting by a miRNA is comparable to that of conserved targets, it is clear that animal miRNAs have had a significant impact on the evolution of most mRNAs.^56^

## Genome-wide probing for microRNA targets

### Computational prediction of microRNA targets

**Figure 2:**
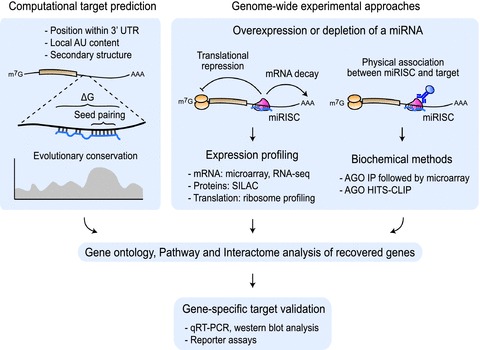
Genome-wide probing for miRNA targets. Computational algorithms for miRNA target prediction rely on a set of established features of miRNA–target interactions, including seed pairing, free energy of the miRNA-target heteroduplex, local AU content, secondary structure, and evolutionary conservation. Genome-wide experimental approaches often start with differential expression of a particular miRNA by overexpression or knockdown. Gene sets for which mRNA or protein levels respond to the perturbation are identified by various expression profiling methods. Alternatively, putative target mRNAs can be directly enriched by Ago immunoprecipitation. Gene ontology and interactome analysis of recovered genes might help elucidate the biological functions of the miRNA. Finally, individual miRNA–target interactions are validated by reporter assays.

Figure 2 summarizes the current genome-wide approaches to miRNA target identification. A collection of algorithms for miRNA target prediction are currently available.^10^,^59^ These algorithms incorporate into their criteria with variable emphasis a set of established features of miRNA-target interactions, such as seed pairing, free energy of the miRNA-target heteroduplex, local AU content, and secondary structure. Evolutionary conservation is often used to filter out noise. Examples of commonly used algorithms include TargetScan, PicTar, and miRanda.^57^,^60^,^61^

Although computational target prediction serves as a reasonable starting point to narrow down the list of candidates, many of the predicted genes are likely to be false positives that should be eliminated by experimental validation. It might be helpful in filtering predictions to consider coexpression of the miRNA and its targets.^62^ Moreover, the algorithms might fail to capture *bona fide* targets that are regulated through noncanonical target sites, such as *RAS* for *let-7* and *E2F2* for miR-24.^36^,^38^

### Gene expression profiling upon differential microRNA expression

Because miRNAs reduce the output of their target genes, one reasonable approach to target identification is to find gene sets for which mRNA or protein levels undergo changes after perturbing the expression of a particular miRNA. The first study of this approach transiently transfected muscle-specific miR-1 and brain-specific miR-124 into HeLa cells that normally do not express them and investigated changes in the cellular transcriptome by microarray analysis.^20^ More than 100 mRNAs were downregulated in each case and over three-quarters of downregulated mRNAs contained seed matches to the transfected miRNA in their 3′ UTRs. Remarkably, the downregulated mRNAs were those for genes that are generally expressed at low levels in the tissue where the transfected miRNA is present as a consequence of selective avoidance.^56^ In a sense, introduction of tissue-specific miRNA shifted the mRNA expression profile of HeLa cells toward that of tissue that normally expresses the miRNA, suggesting that much of the observed targeting is biologically relevant. Knockdown or deletion of endogenous miRNAs has also been employed to achieve differential miRNA expression,^19^,^21^ avoiding potential drawbacks of miRNA overexpression. However, changes in mRNA levels after knockdown were not as great as those after overexpression.^63^

Alternatively, the response of global protein output upon overexpression or depletion of miRNAs can be directly measured by proteomic approaches. Proteomic approaches have an inherent advantage over transcriptome analyses in that they can capture miRNA targets that are primarily regulated at the level of translation. In stable isotope labeling by amino acids in cell culture (SILAC),^64^ proteins are metabolically labeled by cultivating cells in culture medium containing heavy isotope versions of amino acids. Two cell populations, one grown in normal medium and the other SILAC labeled, are mixed in an equal ratio and proteins are analyzed by mass spectrometry, where the relative protein abundance is presented by the ratio of peptide peak intensities. One study used this method to investigate the effect on global protein output of introducing miR-1, miR-124, and miR-181 into HeLa cells and of genetically removing *miR-223* from mouse neutrophils.^22^ Another study used a slight variant of SILAC, pulsed SILAC (pSILAC), where cells are pulse labeled to focus on newly synthesized proteins after the pulse.^23^ In both studies, mRNAs for responsive proteins were enriched in seed matches to the perturbed miRNA.

The major limitation of expression-profiling approaches is that they cannot distinguish between direct and indirect targets, although searching for seed matches in the responsive genes might help draw a distinction. However, they are sometimes useful, when combined with gene ontology and interactome analysis of recovered gene sets, in elucidating biological pathways potentially governed by the given miRNA. For example, mRNAs downregulated upon *let-7* overexpression were enriched in cell cycle and DNA replication pathways, supporting the suspected role of *let-7* as a tumor suppressor.^65^ Similarly, overexpression of miR-24 retrieved gene sets involved in cell cycle progression and DNA repair, and subsequent interactome analysis placed two direct targets of miR-24, *E2F2,* and *MYC*, at the heart of the elaborate network of these genes.^38^ Technical advances such as the development of RNA sequencing (RNA-seq) and further progress in systems biology will facilitate a deeper and more informative analysis of this type of approach.

### Biochemical approaches

Biochemical methods for genome-wide target identification rely on physical association between the miRISC and its target mRNAs. Early attempts were made to immunoprecipitate one of the miRISC components, mainly Ago, and to analyze the associated mRNAs by microarray or sequencing. In two pioneering studies, one in *Drosophila* system and the other in human HEK293S cells, epitope-tagged Ago was ectopically expressed together with an miRNA and immunopurified using an antibody against the epitope tag.^66^,^67^ Microarray profiling of copurified mRNAs showed a significant enrichment for seed matches in their 3′ UTRs or ORFs, highlighting the utility of the technique. However, interactions identified by miRISC immunoprecipitation might not necessarily reflect those that actually occur *in vivo*, as miRISC can dissociate from or nonspecifically associate with mRNAs during cell lysis.^68^

Such false interactions potentially arising from experimental manipulation are minimized in a newly developed technique called crosslinking and immunoprecipitation (CLIP), where cells are first “frozen” by ultraviolet irradiation to crosslink RNA and associated RBPs.^69^ Immunoprecipitation is performed under stringent conditions with an antibody against the RBP of interest, the unbound portion of RNA is digested, and the resulting RBP-protected RNA fragments are analyzed by high-throughput sequencing (the entire procedure is often abbreviated as HITS-CLIP or CLIP-seq). In a variant of CLIP called photoactivatable-ribonucleoside-enhanced CLIP (PAR-CLIP), cells are cultured in the presence of a photo-activatable ribonucleoside analog such as 4-thiouridine, which improves RNA recovery 100- to 1,000-fold compared to conventional CLIP method.^46^ PAR-CLIP also allows to accurately pinpoint the location of the crosslink, as reverse transcription of 4-thiouridine leads to T-C transitions. Recent Ago HITS-CLIP studies have generated a precise genome-wide map of Ago-binding sites in various biological contexts, including mouse brain, *C*. *elegans*, and cultured cells, and have provided compelling data supporting the general features of target sites previously described such as seed pairing and structural accessibility.^39^,^46^,^70^,^71^ Interestingly, Ago-binding sites identified by HITS-CLIP are more often located in ORFs and violate the seed rule more frequently than previously expected, although it is unclear what fraction of these sites represent authentic target sites.

MicroRNA-directed cleavage of target mRNA dictated by near-perfect complementarity is rare in animals, with only few cases reported to date.^34^,^72^ Nonetheless, several efforts were made to search for additional miRNA-directed cleavage events in mammalian transcriptome. They relied on a genome-wide technique called parallel analysis of RNA ends (PARE) or degradome sequencing, where mRNA fragments bearing 5′ monophosphate termini are specifically selected by adaptor ligation and short sequence tags representing their 5′ ends are generated and analyzed by high-throughput sequencing.^73^,^74^ Such mRNA fragments often include 3′ fragments of diverse endonucleolytic cleavage as well as degradation intermediates resulting from 5′ to 3′ exonuclease activities. Reflecting the success of the technique in identifying cleavage targets of miRNAs in plants,^73^,^74^ PARE in cultured cells and tissues of mammals detected several additional mRNAs that are likely to be regulated by miRNA-directed cleavage.^35^,^75^,^76^ The biological significance of these cleavage events remains to be further elucidated. Interestingly, these studies also identified a number of miRNA-independent mRNA cleavage events, such as those catalyzed by another RNAi pathway component Drosha, suggesting the underappreciated role of diverse endonucleolytic processing on transcriptome dynamics.^75^,^76^

## Insights into the mechanism of microRNA-mediated repression

Early insights into the mechanism of miRNA-mediated repression came from studies on the *lin-4* precedent, which demonstrated that *lin-4* represses the translation of the *lin-14* mRNA with little or no effects on its abundance.^77^ Indeed, it became a prevailing notion for a while that animal miRNAs direct translational repression of their target genes without influencing mRNA levels. However, subsequent transcriptomic studies showed that target mRNA abundance also inversely correlates with the level of miRNA, overturning this simplistic notion.^19^–^21^ There followed a number of reports indicating that mRNA decay is an important component of miRNA-mediated repression.^21^,^78^–^81^

Given that both translational repression and mRNA decay are implicated in miRNA-mediated repression, one fundamental question arises: what are the relative contributions of these two mechanisms on repression? To address this issue, one study simultaneously measured mRNA and protein levels in mouse neutrophils isolated from a *miR-233*-knockout mouse.^22^ Notably, changes in mRNA and protein levels compared to wild-type cells strongly correlated, suggesting that most of the regulation was explained by changes in mRNA levels. On the other hand, another study using a similar approach reached a slightly different conclusion.^23^ miR-1 was transfected into HeLa cells and mRNA and protein levels were measured at two different time points after transfection. Interestingly, certain target genes were regulated primarily at the protein level with little change in mRNA levels soon after the transfection (8 h), but they shifted toward greater changes in mRNA levels later (32 h), contributing to the overall correlation between mRNA and protein levels. Taken together, these findings suggest that most of the miRNA-mediated repression accompanies mRNA decay, although a small fraction of targets are repressed at the translational level without apparent changes in mRNA levels.

Subsequent studies have provided further evidence for this mechanistic view of miRNA-mediated repression, employing different readouts for the translational status. For example, one study used polysome profiling to investigate the effects of miR-124 on the occupancy and density of ribosomes on target mRNAs and revealed that mRNA decay accounted for ∼75% of the change observed in protein production.^82^ More recent studies use ribosome profiling to evaluate translational efficiency, where the positions of ribosomes are globally determined with subcodon resolution.^83^ Ribosome profiling in mammalian cells demonstrated that ∼84% of the reduced protein production is attributable to mRNA decay.^84^

Although these studies indicate that mRNA decay provides a major contribution to miRNA-mediated repression, they do not clearly define the initial trigger for the repression, as they primarily focus on the steady states where most of the downstream effects of miRNAs might have been observed. Indeed, one important question that has been intensely debated is whether mRNA decay is a cause or consequence of miRNA-mediated translational repression.^12^ Initial mRNA decay would undoubtedly lead to translational repression, but the contrary can also be supported in the context of intimate coupling of the two processes.^85^ Very recently, two studies examined their ordering in the establishment of miRNA-mediated repression. One study combined ribosome profiling and RNA-seq to monitor the effects of miR-430, a miRNA naturally induced in zebrafish embryos to clear maternal mRNAs, and showed that miR-430 reduces the ribosome occupancy of target mRNAs before causing mRNA decay.^86^ Comparable results were obtained using a controllable reporter system in *Drosophila* S2 cells.^87^ However, whether the same kinetics also apply to other miRNAs in other systems remains to be elucidated.

## Conclusions

Over the last decade, substantial progress has been made in our understanding of how miRNAs interact with their targets, and several key features of those interactions have consequently emerged. However, a genome-wide “snapshot” of miRNA–target interactions clearly indicates that those features can explain only a fraction of all targeting events. A deeper understanding of miRNA–target interactions is necessary to develop more reliable approaches to miRNA target identification.

## References

[1] Lee RC, Feinbaum RL, Ambros V (1993). The C. elegans heterochronic gene lin-4 encodes small RNAs with antisense complementarity to lin-14. Cell.

[2] Wightman B, Ha I, Ruvkun G (1993). Posttranscriptional regulation of the heterochronic gene lin-14 by lin-4 mediates temporal pattern formation in C. elegans. Cell.

[3] Reinhart BJ (2000). The 21-nucleotide let-7 RNA regulates developmental timing in Caenorhabditis elegans. Nature.

[4] Pasquinelli AE (2000). Conservation of the sequence and temporal expression of let-7 heterochronic regulatory RNA. Nature.

[5] Lagos-Quintana M (2001). Identification of novel genes coding for small expressed RNAs. Science.

[6] Lau NC (2001). An abundant class of tiny RNAs with probable regulatory roles in Caenorhabditis elegans. Science.

[7] Lee RC, Ambros V (2001). An extensive class of small RNAs in Caenorhabditis elegans. Science.

[8] Kozomara A, Griffiths-Jones S (2011). miRBase: integrating microRNA annotation and deep-sequencing data. Nucleic Acids Res.

[9] Kim VN, Han J, Siomi MC (2009). Biogenesis of small RNAs in animals. Nat. Rev. Mol. Cell. Biol.

[10] Bartel DP (2009). MicroRNAs: target recognition and regulatory functions. Cell.

[11] Krol J, Loedige I, Filipowicz W (2010). The widespread regulation of microRNA biogenesis, function and decay. Nat. Rev. Genet.

[12] Huntzinger E, Izaurralde E (2011). Gene silencing by microRNAs: contributions of translational repression and mRNA decay. Nat. Rev. Genet.

[13] Pasquinelli AE (2012). MicroRNAs and their targets: recognition, regulation and an emerging reciprocal relationship. Nat. Rev. Genet.

[14] Chalfie M, Horvitz HR, Sulston JE (1981). Mutations that lead to reiterations in the cell lineages of C. elegans. Cell.

[15] Ambros V (1989). A hierarchy of regulatory genes controls a larva-to-adult developmental switch in C. elegans. Cell.

[16] Lim LP (2003). The microRNAs of Caenorhabditis elegans. Genes Dev.

[17] Lai EC (2002). Micro RNAs are complementary to 3′ UTR sequence motifs that mediate negative post-transcriptional regulation. Nat. Genet.

[18] Lewis BP (2003). Prediction of mammalian microRNA targets. Cell.

[19] Krutzfeldt J (2005). Silencing of microRNAs in vivo with ‘antagomirs’. Nature.

[20] Lim LP (2005). Microarray analysis shows that some microRNAs downregulate large numbers of target mRNAs. Nature.

[21] Giraldez AJ (2006). Zebrafish MiR-430 promotes deadenylation and clearance of maternal mRNAs. Science.

[22] Baek D (2008). The impact of microRNAs on protein output. Nature.

[23] Selbach M (2008). Widespread changes in protein synthesis induced by microRNAs. Nature.

[24] Haley B, Zamore PD (2004). Kinetic analysis of the RNAi enzyme complex. Nat. Struct. Mol. Biol.

[25] Ameres SL, Martinez J, Schroeder R (2007). Molecular basis for target RNA recognition and cleavage by human RISC. Cell.

[26] Wang Y (2008). Structure of the guide-strand-containing argonaute silencing complex. Nature.

[27] Schirle NT, Macrae IJ (2012). The crystal structure of Human Argonaute2. Science.

[28] Elkayam E (2012). The structure of Human Argonaute-2 in complex with miR-20a. Cell.

[29] Lewis BP, Burge CB, Bartel DP (2005). Conserved seed pairing, often flanked by adenosines, indicates that thousands of human genes are microRNA targets. Cell.

[30] Nielsen CB (2007). Determinants of targeting by endogenous and exogenous microRNAs and siRNAs. RNA.

[31] Ma JB (2005). Structural basis for 5′-end-specific recognition of guide RNA by the A. fulgidus Piwi protein. Nature.

[32] Parker JS, Roe SM, Barford D (2005). Structural insights into mRNA recognition from a PIWI domain-siRNA guide complex. Nature.

[33] Wang Y (2008). Structure of an argonaute silencing complex with a seed-containing guide DNA and target RNA duplex. Nature.

[34] Yekta S, Shih IH, Bartel DP (2004). MicroRNA-directed cleavage of HOXB8 mRNA. Science.

[35] Shin C (2010). Expanding the microRNA targeting code: functional sites with centered pairing. Mol. Cell.

[36] Johnson SM (2005). RAS is regulated by the let-7 microRNA family. Cell.

[37] Tay Y (2008). MicroRNAs to Nanog, Oct4 and Sox2 coding regions modulate embryonic stem cell differentiation. Nature.

[38] Lal A (2009). miR-24 Inhibits cell proliferation by targeting E2F2, MYC, and other cell-cycle genes via binding to “seedless” 3′UTR microRNA recognition elements. Mol. Cell.

[39] Chi SW (2009). Argonaute HITS-CLIP decodes microRNA-mRNA interaction maps. Nature.

[40] Chi SW, Hannon GJ, Darnell RB (2012). An alternative mode of microRNA target recognition. Nat. Struct. Mol. Biol.

[41] Grimson A (2007). MicroRNA targeting specificity in mammals: determinants beyond seed pairing. Mol. Cell.

[42] Gu S (2009). Biological basis for restriction of microRNA targets to the 3′ untranslated region in mammalian mRNAs. Nat. Struct. Mol. Biol.

[43] Tian B (2005). A large-scale analysis of mRNA polyadenylation of human and mouse genes. Nucleic Acids Res.

[44] Sandberg R (2008). Proliferating cells express mRNAs with shortened 3′ untranslated regions and fewer microRNA target sites. Science.

[45] Mayr C, Bartel DP (2009). Widespread shortening of 3′UTRs by alternative cleavage and polyadenylation activates oncogenes in cancer cells. Cell.

[46] Hafner M (2010). Transcriptome-wide identification of RNA-binding protein and microRNA target sites by PAR-CLIP. Cell.

[47] Forman JJ, Legesse-Miller A, Coller HA (2008). A search for conserved sequences in coding regions reveals that the let-7 microRNA targets Dicer within its coding sequence. Proc. Natl. Acad. Sci. U. S. A.

[48] Kertesz M (2007). The role of site accessibility in microRNA target recognition. Nat. Genet.

[49] Long D (2007). Potent effect of target structure on microRNA function. Nat. Struct. Mol. Biol.

[50] Bhattacharyya SN (2006). Relief of microRNA-mediated translational repression in human cells subjected to stress. Cell.

[51] Mukherjee N (2011). Integrative regulatory mapping indicates that the RNA-binding protein HuR couples pre-mRNA processing and mRNA stability. Mol. Cell.

[52] Kedde M (2007). RNA-binding protein Dnd1 inhibits microRNA access to target mRNA. Cell.

[53] Saetrom P (2007). Distance constraints between microRNA target sites dictate efficacy and cooperativity. Nucleic Acids Res.

[54] Didiano D, Hobert O (2006). Perfect seed pairing is not a generally reliable predictor for miRNA-target interactions. Nat. Struct. Mol. Biol.

[55] Garcia DM (2011). Weak seed-pairing stability and high target-site abundance decrease the proficiency of lsy-6 and other microRNAs. Nat. Struct. Mol. Biol.

[56] Farh KK (2005). The widespread impact of mammalian microRNAs on mRNA repression and evolution. Science.

[57] Krek A (2005). Combinatorial microRNA target predictions. Nat. Genet.

[58] Stark A (2005). Animal microRNAs confer robustness to gene expression and have a significant impact on 3′UTR evolution. Cell.

[59] Thomas M, Lieberman J, Lal A (2010). Desperately seeking microRNA targets. Nat. Struct. Mol. Biol.

[60] Friedman RC (2009). Most mammalian mRNAs are conserved targets of microRNAs. Genome Res.

[61] John B (2004). Human microRNA targets. PLoS Biol.

[62] Ritchie W, Flamant S, Rasko JE (2009). Predicting microRNA targets and functions: traps for the unwary. Nat. Methods.

[63] Linsley PS (2007). Transcripts targeted by the microRNA-16 family cooperatively regulate cell cycle progression. Mol. Cell. Biol.

[64] Mann M (2006). Functional and quantitative proteomics using SILAC. Nat. Rev. Mol. Cell. Biol.

[65] Johnson CD (2007). The let-7 microRNA represses cell proliferation pathways in human cells. Cancer Res.

[66] Easow G, Teleman AA, Cohen SM (2007). Isolation of microRNA targets by miRNP immunopurification. RNA.

[67] Karginov FV (2007). A biochemical approach to identifying microRNA targets. Proc. Natl. Acad. Sci. U. S. A.

[68] Mili S, Steitz JA (2004). Evidence for reassociation of RNA-binding proteins after cell lysis: implications for the interpretation of immunoprecipitation analyses. RNA.

[69] Ule J (2003). CLIP identifies Nova-regulated RNA networks in the brain. Science.

[70] Zisoulis DG (2010). Comprehensive discovery of endogenous Argonaute binding sites in Caenorhabditis elegans. Nat. Struct. Mol. Biol.

[71] Leung AK (2011). Genome-wide identification of Ago2 binding sites from mouse embryonic stem cells with and without mature microRNAs. Nat. Struct. Mol. Biol.

[72] Davis E (2005). RNAi-mediated allelic trans-interaction at the imprinted Rtl1/Peg11 locus. Curr. Biol.

[73] German MA (2008). Global identification of microRNA-target RNA pairs by parallel analysis of RNA ends. Nat. Biotechnol.

[74] Addo-Quaye C (2008). Endogenous siRNA and miRNA targets identified by sequencing of the Arabidopsis degradome. Curr. Biol.

[75] Karginov FV (2010). Diverse endonucleolytic cleavage sites in the mammalian transcriptome depend upon microRNAs, Drosha, and additional nucleases. Mol. Cell.

[76] Bracken CP (2011). Global analysis of the mammalian RNA degradome reveals widespread miRNA-dependent and miRNA-independent endonucleolytic cleavage. Nucleic Acids Res.

[77] Olsen PH, Ambros V (1999). The lin-4 regulatory RNA controls developmental timing in Caenorhabditis elegans by blocking LIN-14 protein synthesis after the initiation of translation. Dev. Biol.

[78] Rehwinkel J (2005). A crucial role for GW182 and the DCP1:DCP2 decapping complex in miRNA-mediated gene silencing. RNA.

[79] Behm-Ansmant I (2006). mRNA degradation by miRNAs and GW182 requires both CCR4:NOT deadenylase and DCP1:DCP2 decapping complexes. Genes Dev.

[80] Wu L, Fan J, Belasco JG (2006). MicroRNAs direct rapid deadenylation of mRNA. Proc. Natl. Acad. Sci. U. S. A.

[81] Eulalio A (2009). Deadenylation is a widespread effect of miRNA regulation. RNA.

[82] Hendrickson DG (2009). Concordant regulation of translation and mRNA abundance for hundreds of targets of a human microRNA. PLoS Biol.

[83] Ingolia NT (2009). Genome-wide analysis in vivo of translation with nucleotide resolution using ribosome profiling. Science.

[84] Guo H (2010). Mammalian microRNAs predominantly act to decrease target mRNA levels. Nature.

[85] Schwartz DC, Parker R (1999). Mutations in translation initiation factors lead to increased rates of deadenylation and decapping of mRNAs in Saccharomyces cerevisiae. Mol. Cell. Biol.

[86] Bazzini AA, Lee MT, Giraldez AJ (2012). Ribosome profiling shows that miR-430 reduces translation before causing mRNA decay in zebrafish. Science.

[87] Djuranovic S, Nahvi A, Green R (2012). miRNA-mediated gene silencing by translational repression followed by mRNA deadenylation and decay. Science.

